# Using standardized patient encounters to teach longitudinal continuity of care in a family medicine clerkship

**DOI:** 10.1186/s12909-016-0733-y

**Published:** 2016-08-17

**Authors:** Bonnie M. Vest, Abigail Lynch, Denise McGuigan, Timothy Servoss, Karen Zinnerstrom, Andrew B. Symons

**Affiliations:** 1 Department of Family Medicine, University at Buffalo, 77 Goodell Street, Suite 220, Buffalo, NY 14203 USA; 2 Department of Anthropology, University at Buffalo, 380 MFAC, Buffalo, NY 14228 USA; 3 Department of Family Medicine, University at Buffalo, 202 Farber Hall, Buffalo, NY 14214 USA; 4 Department of Psychology, Canisius College, 2001 Main Street, Buffalo, NY 14208 USA; 5 Clinical Competency Center, Jacobs School of Medicine and Biomedical Sciences, University at Buffalo, 211 Cary Hall, Buffalo, NY 14214 USA

**Keywords:** Continuity of care, Family medicine, Medical education, Standardized patient encounters

## Abstract

**Background:**

Despite demonstrated benefits of continuity of care, longitudinal care experiences are difficult to provide to medical students. A series of standardized patient encounters was developed as an innovative curricular element to address this gap in training for medical students in a family medicine clerkship. The objective of this paper is to describe the development and implementation of the curriculum, evaluate the effectiveness of the curriculum for increasing student confidence around continuity of care and chronic disease management, and explore student opinions of the value of the experience.

**Methods:**

The encounters simulate continuity of care in typical family medicine practice over four standardized patient visits, providing students with experience in longitudinal relationships, ongoing management of chronic and acute conditions, lifestyle counseling, and the use of an electronic medical record. Perceptions of the curriculum were obtained using a pre-post survey asking students to self-rate experience and confidence in continuity relationships, chronic disease management, and lifestyle counseling. Students were also asked about the overall effectiveness of the encounters for simulating family practice and continuity of care. Open-ended comments were gathered through weekly reflection papers submitted by the students.

**Results:**

Of 138 third-year medical students, 137 completed the pre-survey, 126 completed the post-survey, and 125 (91%) completed both the pre- and the post-survey. Evaluation results demonstrated that students highly valued the experience. Complete confidence data for 116 students demonstrated increased confidence pre-post (t(115) = 14.92, *p* < .001) in managing chronic disease and establishing relationships. Open-ended comments reflected how the experience fostered appreciation for the significance of patient-doctor relationships and continuity of care.

**Conclusions:**

This curriculum offers a promising approach to providing students with continuity of care experience. The model addresses a general lack of training in continuity of care in medical schools and provides a standardized method for teaching chronic disease management and continuity relationships.

**Electronic supplementary material:**

The online version of this article (doi:10.1186/s12909-016-0733-y) contains supplementary material, which is available to authorized users.

## Background

It is well-established that longitudinal care from a primary care physician improves quality and efficiency of patient care. Patients report higher satisfaction in the context of their care when engaged in a long-term relationship with one healthcare provider [1–3]. Better patient health outcomes have also been associated with continuity of care [2, 4]. In addition, longitudinal care promotes cost efficacy; for instance, by decreasing the number of hospital and/or emergency department visits [5–7].

Though training and experience in longitudinal care supports student interest in primary care careers [8–10], the structure of medical education makes it difficult for large numbers of students to experience providing continuity of care. Standardized patient encounters usually simulate “one-time” interactions with no opportunity for the student to consider the continuity of care that would be provided to that patient in a true outpatient setting, especially in a primary care practice. In terms of clinical experiences, the short length of most clerkships simply doesn’t allow enough time for students to get a full picture of continuity of care and longitudinal patient-physician relationships [11, 12]. Students rarely, if ever, have the opportunity to see the same patient more than once in a clinical setting.

Numerous aspects of traditional academic medical center organization and culture have been noted as barriers to improving continuity of care experience for medical students. Examples of these barriers include: under-investment in infrastructure, lack of investment in ambulatory care facilities, the necessity to comply with officially mandated curricula, difficulty in coordinating among various faculty and departments, national shortages of preceptors and increasing medical school class sizes [13–15].

Despite these difficulties, many medical schools have implemented programs to enhance students’ continuity of care experiences. One solution which addresses this issue, but drastically changes the structure of medical school curriculum, is the “longitudinal integrated clerkship.” In longitudinal integrated clerkships, the normal curriculum structure of 6- to 8-weeks of “block clerkships”[16] is replaced by an integrated clerkship, occurring for a median of 40 weeks, in which students continually experience the different required core competencies (family medicine, surgery, etc.) [17].

Other universities have developed alternative approaches to providing continuity of care experience. In Dundee University Medical School’s program, “The Patient Journey,” students are assigned to a patient and make home visits over a period of 3 years; however, they cannot examine or treat the patient, so they are limited to gaining continuity experience in a social context [18]. The University of Louisville recently developed a longitudinal standardized patient model for medical students in preclinical years. Students participate in a continuity relationship with one standardized patient on multiple occasions throughout their first 2 years of school [19].

At our institution, the family medicine clerkship director (AS) proposed we complement and support existing clinical experiences by developing an innovative curricular element using a series of standardized patient encounters to simulate continuity of care experience. As part of a series of initiatives to increase student interest in primary care careers, the curriculum was intended to allow students to experience continuity of care and develop an appreciation of its’ value for both patients and physicians. All third-year medical students participate in these encounters as part of their family medicine clerkship, allowing them to experience patient continuity, practice physical exam skills, manage multiple coexisting chronic diseases, diagnose, formulate a treatment plan, and utilize an electronic medical record (EMR), without requiring extensive modification of the medical school curriculum.

The objective of this paper is to describe the development and implementation of this curricular innovation, evaluate the effectiveness of the curriculum for increasing student confidence around continuity of care and chronic disease management, and explore student opinions of the value of the experience.

## Methods

### Curriculum description

The longitudinal standardized patient encounters take place during the third-year family medicine clerkship. All students are required to participate in these encounters.

Students encounter the same standardized patient for four consecutive weeks, simulating a 6-month time period. Students interact with the standardized patient independently. An easy-to-navigate EMR program (Simple EMR, Copyright© 2008–2015, digitalcairo.com) was used and students brought laptops with the EMR program into the exam room for patient visits. Having the EMR in the exam room provided students the opportunity to practice integrating usage into a patient visit and make decisions about when to make notes, when to share the screen with the patient, and how to position themselves during the encounter. Elements of the EMR were populated with relevant patient data and updated weekly to reflect progression of the patient case. Students use the EMR during the patient encounter to review lab results with the patient, view patient medical history, and complete progress notes.

There are approximately 20 students rotating through each clerkship. The encounters with the standardized patients occur in shifts of five. All encounters are completed on the same day, during regularly scheduled didactic time for the clerkship, so students do not miss time in the clinical setting. Each group of five students is assigned to a time slot for their standardized patient visit. Students arrive a few minutes prior to their scheduled time to review the scenario and the EMR notes. They then conduct the encounter and spend approximately 20 min afterwards to complete and print their notes in the EMR. The entire group reconvenes after all the encounters conclude for debriefing. Three of the encounters occur in our clinical competency center, which simulates an outpatient clinic. One encounter occurs in our simulation center, which simulates a hospital emergency department room (the EMR program was not used for the hospital-based encounter, as it would be unlikely for the same EMR to be used in an outpatient and hospital setting).

Five standardized patients receive two hours of training before each encounter. Standardized patients were recruited from the university’s existing standardized patient program. In addition, we incorporated five administrative staff to coordinate the encounters; one to assist students with their EMRs in the computer lab; two to facilitate the logistics of the encounters, such as timing the visits and recording the interactions; and two employees of the standardized patient program to review the scenarios with the standardized patients and monitor the encounters.

Unlike traditional standardized patient encounters, students are not graded on performance. Rather, the emphasis is on allowing the student to develop clinical skills and patient relationships in a low-pressure environment. The standardized patient offers the student feedback on their interpersonal communication skills and professionalism at the end of each session, except the “emergency room” session (due to the logistics of that session). The standardized patients also complete a brief on-line checklist and provide open-ended comments regarding communication skills and professionalism which are available for students to review.

After all students complete their encounter, a faculty member debriefs the entire group starting with students’ impressions of the encounter. Other discussion topics are outlined in Table 1 and include elements of clinical care, time management and the doctor-patient relationship. Since it would be logistically difficult to create a follow-up scenario for each student reflecting their own specific medical decision-making, we created a unified progress note which reflects a “reasonable” management plan for the patient and serves as a basis for the follow-up visit. Students receive this progress note at the end of the debriefing. We acknowledge in the debriefing that there are many acceptable approaches to managing the patient’s care, and the creation of this “standard progress note” is simply a logistical necessity given the structure of the program. Students submit a brief written reflection on each encounter prior to the following week’s session. In their reflections students are asked to comment on how they felt about the encounter, what they felt they did well and what they need to improve upon, and their overall impressions of the whole exercise. Student reflections incorporate insights gained in the debriefing session and discussions with preceptors and clerkship faculty, as well as any independent reading undertaken by the student. Faculty who teach in the clerkship review these reflections. Each of the four scenarios and the corresponding debriefing topics are summarized in Table 1. Family medicine faculty, as well as faculty from the school’s clinical competency center, developed the scenarios.

**Table 1:** Scenarios and Debriefing Discussion Topics for the Standardized Patient Encounters

	*Scenario Synopsis*	*Debriefing Discussion Topics*
Scenario 1	The student meets the patient for the first time in a 30 min encounter. The patient is already established in the practice, though their physician retired this year. This is their initial visit with the new physician (i.e. the student). The EMR is populated with the patient’s history as well as labs that were completed one week prior to the visit. Last year, the patient’s family physician told her she had “pre-diabetes”, mildly elevated cholesterol, and hypertension. Diet and exercise recommendations were discussed last year. She smokes. Labs done one week ago show hemoglobin A1C 8.1, low-density lipoprotein cholesterol 140. Body Mass Index is 32. Blood pressure is 150/90 and consistent with home measurements.	• Importance of continuity of care • Establishment of rapport with new patient • Effective use of the EMR • Medical management of multiple coexisting chronic diseases (diabetes, hypertension, hyperlipidemia) • Lifestyle intervention (weight-loss, smoking cessation) • Appropriate interprofessional referral in management of chronic disease (i.e. diabetes educator, dietician, etc.) • Appropriate follow-up • Time management • Review of the standard progress note to be used as the basis for the next visit
Scenario 2	This is a three-month follow up visit. The patient started lisinopril and metformin after the last visit. Her diabetes control and blood pressure have improved. She met with the diabetes educator and incorporated some dietary changes which have led to modest weight loss. She is still reluctant to take a statin medication due to things she has seen on television, and reports of some family members who developed joint and muscle pain on these medications. She continues to smoke. Today she complains of acute knee pain (consistent with a lateral collateral ligament sprain) and chronic shoulder pain (consistent with rotator cuff tendonitis).	• Acknowledging success in addressing chronic disease • Negotiating medication recommendations and patient perceptions of potential side effects • Acute care in the setting of a routine follow-up visit • Appropriate management of musculoskeletal complaints • Relationship building and continuity of care • Review of the standard progress note to be used as the basis for the next visit
Scenario 3	This visit occurs in a simulated hospital room. Patient presents to emergency department with chest pain. Although there is an exertional component to the chest pain, and the patient has risk factors for coronary artery disease, it is also noted that the pain is epigastric. The patient has been taking ibuprofen at an anti-inflammatory dose since the last office visit 6 weeks ago. Students are asked to perform a focused history and physical. When they are ready to order diagnostics or therapeutics, they leave the room and discuss the case with a faculty member. We have prepared results for common diagnostics ordered when a patient with risk factors presents with chest pain (e.g. electrocardiogram, chest x-ray, cardiac enzymes, stress test results, etc.). Once the student orders the diagnostics and therapeutics, they return to the patient room after a simulated elapse of 24 hours. The cardiac work-up is negative of ischemia, and, on further questioning, it appears that the pain may be related to continued use of non-steroidal anti-inflammatories.	• Inpatient care in family medicine • Assessment of cardiac risk and work-up of acute chest pain • Differential diagnosis of chest pain *•* Review of the discharge summary to be used as the basis for the next visit
Scenario 4	This is a post hospitalization outpatient follow-up visit. Although it was established that the “chest pain” was likely gastritis from non-steroidal use, the episode frightened the patient, and she wants to attempt smoking cessation, and consider a statin medication.	• Hospital follow-up • Medication reconciliation • Motivational interviewing (smoking cessation) • Routine health maintenance and screening • Overall standardized patient feedback

We piloted the curriculum in the spring of 2013 with four students from each of three clerkship rotations. After each session, students, the standardized patients, and faculty met to discuss the content and logistics of the encounter. Using this input, we modified logistics and the scenarios. The curriculum was fully implemented at the start of the 2013–2014 academic year.

### Data collection

In order to obtain student feedback on the perceived value of the experience, we developed a pre/post survey (see Additional files 1 and 2) and analyzed student reflection papers. In the pre-survey we asked students about their experience and confidence level at baseline (i.e. the beginning of the family medicine clerkship) with providing continuity of care; developing rapport with a patient; clinical disease management of diabetes, hypertension and hyperlipidemia; and providing lifestyle counseling related to diet, exercise and smoking cessation. Students rated their experience and confidence on a 4-point scale ranging from “no experience/confidence” to “lots of experience/very confident.” The items formed a scale with excellent reliability (alpha = 0.92). We also asked students how many times they had encountered the same patient more than once in an outpatient setting and which clerkship rotations they had already completed.

The post-survey repeated the same questions about confidence and then asked students how they would rate the encounters as a simulation of typical family medicine practice, as a simulation of chronic disease management, as a simulation of continuity of care, as representative of the type of patients seen in family medicine practice, and as an important contribution to their medical education. Students were asked to complete a range of open-ended questions asking what they liked about the encounters and what changes they would recommend.

We administered the surveys electronically via E*Value^TM^ Healthcare Education Solutions (Advanced Informatics LLC), an internet-based program that maintains accreditation standards related to student curriculum and course evaluations, electronic portfolios, procedure tracking, scheduling, coursework management, time tracking, curriculum mapping, outcomes management, and performance reporting. Surveys appeared as optional items for students to complete at the beginning and end of their clerkship rotation. Students received an information sheet about the curriculum evaluation as a preface to the survey, and consent was indicated by submission of the survey. De-identified responses were extracted by clerkship staff into an Excel spreadsheet and shared with us for analysis. We conducted surveys with all of the eight clerkship groups for the 2013–2014 school year. Students emailed reflection papers to clerkship staff at the end of each standardized patient encounter. Staff removed student names and provided us with the full text of the reflections for analysis. For this paper, only the final reflection was used, as it provided the student the opportunity to reflect back on the value of the entire experience. The Institutional Review Board (IRB) approved a waiver of consent for the review of de-identified reflection papers, as they were collected for purposes other than research and were anonymous. The University at Buffalo Social and Behavioral Sciences Institutional Review Board determined that the evaluation of this curriculum qualified for an exemption (IRB Study #444902–1).

### Data analysis

We coded quantitative survey responses in Excel and imported them into SPSS 22 (Armonk, NY) for analysis. Paired-samples t-tests were based on matched pre- and post-test data from the individual students. In an effort to conduct a conservative analysis, we assessed changes in individual item scores using the Wilcoxon signed rank test, as difference scores for the individual items are less likely to adhere to the distributional assumptions of the paired-samples t-test.

We analyzed the open-ended qualitative responses and reflection papers using a thematic content analysis approach [20]. Responses were grouped based upon common content related to a particular theme.

## Results

Of 138 third-year medical students, 137 completed the pre-survey, 126 completed the post-survey, and 125 (91%) completed both the pre- and the post-survey.

Overall, students indicated limited previous experience with seeing the same patient in an outpatient setting more than once (43.7% indicated ≤3 continuity experiences prior to the family medicine clerkship). While the reported numbers were higher for the family medicine clerkship, they still demonstrate opportunity for improvement in exposure of medical students to longitudinal patient relationships (Table 2).

**Table 2:** Number of times students reported seeing the same patient more than once

How many times did you see the same patient more than once in an outpatient setting?				
	Never	Once	2–3 Times	More than 3 times
Medical School (pre Family Medicine) (*n* = 137)	16% (22)	27.7% (38)	44.5% (61)	11.7% (16)
Family Medicine Clerkship (*n* = 126)	7.9% (10)	18.3% (23)	57.9% (73)	15.9% (20)

Students rated the curriculum highly across all the surveyed domains (Table 3).

**Table 3:** Student Ratings (*n* = 125) of the curriculum

On a scale of 1–5 (1 = Poor, 5 = Excellent) how would you rate the curriculum:	
Measure	Mean Score (Standard Deviation (SD))
As a simulation of typical family medicine practice	4.55 (.63)
As a simulation of chronic disease management	4.56 (.54)
As a simulation of continuity of care	4.63 (.56)
As representative of the type of patient seen in family medicine practice	4.47 (.70)
As an important contribution to your medical education	4.62 (.65)

Of the 125 students who completed both the pre- and the post-survey, 116 had complete responses to all of the confidence items. The responses of these 116 students were used to assess changes in confidence after experiencing the curriculum. Students indicated increased confidence in chronic disease management, lifestyle counseling, and developing a continuity relationship with patients as a result of participation in the encounters. A composite confidence score equal to the sum of student responses to each of the confidence questions was created for pre- and post-tests. The means were compared, and demonstrated a significant increase in total confidence pre/post (t(115) = 14.92, *p* < .001). This improvement in confidence from pre to post was consistent across all eight clerkship groups. Using a series of Wilcoxon Signed Ranks Tests, the individual items also all showed a significant increase in confidence from pre/post. The effect size of the difference in total confidence (d = 1.44) as well as the individual item differences were large (See Table 4).

**Table 4:** Medical student (*n* = 116) self-reported increase in confidence* across all items, pre-post

Measure	Pre-Test Mean (SD)	Post-Test Mean (SD)	Mean Difference (Post-Pre)	95% Confidence Interval of Difference	*P* value	Effect Size
Providing continuity of care	1.75 (.62)	2.47 (.50)	0.72	.84, .61	<.001	1.17
Establishing rapport with patients	2.32 (.56)	2.74 (.44)	0.42	.53, .31	<.001	0.74
Expressing empathy	2.33 (.64)	2.67 (.49)	0.34	.45, .22	<.001	0.53
Management of diabetes	1.64 (.64)	2.43 (.54)	0.79	.91, .67	<.001	1.23
Management of hypertension	1.72 (.60)	2.52 (.53)	0.80	.92, .67	<.001	1.32
Management of hyperlipidemia	1.64 (.64)	2.45 (.53)	0.81	.93, .69	<.001	1.27
Lifestyle counseling on smoking	1.82 (.65)	2.48 (.56)	0.66	.78, .53	<.001	1.01
Lifestyle counseling on diet	1.79 (.69)	2.48 (.58)	0.69	.82, .57	<.001	1.01
Lifestyle counseling on exercise	1.85 (.68)	2.54 (.57)	0.69	.82, .57	<.001	1.02
Demonstrating whole person care	1.72 (.62)	2.50 (.56)	0.78	.92, .65	<.001	1.28
Understanding how a patient’s context impacts their health	1.84 (.58)	2.56 (.53)	0.72	.84, .59	<.001	1.24
Using an EMR in conjunction with a patient visit	1.84 (.70)	2.43 (.60)	0.59	.73, .44	<.001	0.84

*Confidence was scored on a scale from 0 (not at all confident) to 3 (very confident). *P* values for the change in individual items are based on the Wilcoxon signed rank test. Effect sizes calculated as (post-test mean- pre-test mean)/ pre-test standard deviation

Analysis of the reflection papers and the open-ended survey questions revealed six main themes: 1) an appreciation of “independence” and the opportunity to “be a doctor”; 2) a recognition of the significance of continuity of care; 3) an appreciation for patient relationships; 4) an appreciation for primary care as a career; 5) reflection on what was learned; and 6) the overall value of the curriculum. Themes and illustrative comments are presented in Table 5.

**Table 5:** Student Comments on the Curriculum from Reflection Papers and Post-Survey

Theme	Illustrative Comments
Appreciated “independence”/ “being a doctor”	“I liked having to ‘come up with everything’. Sometimes in the rush, it’s hard for attendings to have the time to let students talk through/present a whole plan. I think being able to do that was really useful.”
	"The whole experience was great for me. At the end of these encounters I didn’t feel like I was ‘playing doctor,’ I felt as if I was the doctor.”
Recognized the significance of continuity of care	“I feel continuity is the best part about family medicine and having the opportunity to follow patients and their families over years is a very valuable and appealing aspect of family medicine, not only for personal relationships but also to deliver the best care possible to your patients.”
	“The [curriculum] enhanced the outpatient experience somewhat by allowing us to experience a continuity of care that we would normally not be able to see in the time given for the clerkship….I never really understood the significance of it until I started working in an outpatient setting. Sometimes at the office, I would find myself wondering how nice it would be to see a patient improve after I saw him, and then realize that I would not even be around for his follow-up. The [curriculum] let me see the same patient over a period of time and vary care based on their needs, and I appreciate that all the more now knowing how rare it is for that to happen as a medical student.”
	“As I’ve gone through this exercise I’ve found it easier and easier to talk to and connect with my patient-this is the first time that I’ve personally felt how rewarding continuity of care can be. Not only did I feel like my patient trusted and liked me, I also felt confident in my ability to care for her.”
Appreciation for patient relationships	“I think that was the best part of the exercise, developing a relationship of trust and friendship with the patient.”
	“I liked making a connection and forming a relationship with my patient…The more you know them and the more comfortable they feel with you, the easier it will be to treat them and keep them healthy.”
Appreciation for primary care as a career	“Over all this experience was a wonderful example of continuity of care. I can honestly say that I have never had an interest in pursuing a career in family medicine before this rotation. After this experience and learning more about the vast skills family medicine practitioners can practice regularly I can see myself becoming seriously interested in this field.”
Reflection on what was learned	“It helped me understand how to become comfortable with the patients and how to really listen to their needs.”
	“The most important thing in my opinion was the practice in establishing a relationship, managing medical problems, and being able to multitask to deal with many issues as once.”
	“I feel this experience has taught me a great deal about caring for and treating a complex patient.”
Overall value of curriculum	“I enjoyed taking care of the same patient in multiple visits. It was a unique experience that I am not accustomed to.”
	“The [curriculum] is one of the best parts of this rotation.”
	“This experience will be one that I can take far beyond the scope of this clerkship.”

Overall, students felt this model had provided them with valuable experiences that enhanced their education. Reflections indicated a deeper appreciation for the significance of continuity relationships and how rewarding those relationships can be. Students also reported that they gained skills and knowledge in the management of multiple chronic conditions, relationship building, and EMR use, which they felt could be applied to their real world clinical experience. Students especially appreciated being able to independently manage patient care and make clinical decisions in an environment where they were not graded and felt able to make mistakes and learn from them.

Many students indicated that they had no suggestions for improvement of the experience. Those who did respond desired more emergency room and/or hospital-based scenarios and an expanded longitudinal curriculum with additional content or more encounters to allow for greater variety. Some students did not like that the follow-up visits were standardized, instead of reflecting their individual decision-making. One student commented that students should be able to follow-up on their own recommendations “*to see how our personal management style affects the patient’s health.”* Other students indicated that they would appreciate more feedback from faculty and the patient. Some students felt that the standardized patients were too willing to make lifestyle changes compared to real patients.

## Discussion

Evaluation of the curriculum demonstrated that a series of longitudinal standardized patient encounters was well-liked by students, increased student confidence in chronic disease management and continuity relationships, and impacted student perceptions of continuity of care and primary care practice.

Medical schools, including ours, have recognized the benefits of using standardized patients in standardized observed clinical settings [21, 22]. However, these exercises generally simulate “one-time” encounters, with no opportunity for the student to consider the continuity of care that would be provided to that patient in primary care practice. We addressed this by developing a set of standardized patient encounters to provide third-year medical students with experience in continuity of care. Our survey results demonstrate that the curriculum was well-liked by students, who rated it highly as a simulation of continuity of care and typical family medicine practice. Students also indicated that they felt the curriculum was relevant and useful for their clinical rotations. Students were especially appreciative of the opportunity to make independent clinical decisions and reflect on how those decisions affected the patient. They enjoyed seeing the same patient repeatedly and learning how to manage patient encounters in a continuity relationship. Student reflections and comments on the post-survey indicated an appreciation for this experience, and a high perceived value as an addition to their medical education, especially because it allowed them to develop relationships with patients and experience first-hand the value of continuity of care.

As of March 2015, more than 30 schools worldwide are listed as having some sort of longitudinal integrated clerkship program [23]. These programs, however, tend to be logistically complex and costly, with money needed for “increased student travel, faculty travel, faculty development, additional staff needed to handle the increased logistical complexity, and other factors” [16, 17, 24]. Longitudinal curricular models are often for select groups of students, who elect for a continuity or primary care track [25–30]. Our model addresses some of these problems, allowing all students to experience continuity of care without requiring substantial changes in the structure of the medical school curriculum.

The University of Louisville implemented a similar longitudinal standardized patient project [19]. Students see the same standardized patient across 19 encounters. This program was instituted for first- and second-year students to help overcome the time constraints associated with obtaining patient histories at each standardized patient visit and to provide students time to focus on communication skills for each session. Our model differs slightly, in that it allows students in their clinical years to experience and practice clinical management of chronic diseases in a longitudinal setting. Our model also incorporates the use of an EMR, providing students with experience in integrating EMR usage into the patient visit.

Even in medical schools that already provide continuity experiences, this curricular element offers a benefit in the form of a *standardized* opportunity to teach lessons related to chronic disease management and continuity relationships. This standardized model provides benefits that are not always available in the preceptor’s office, including: allowing students to experience making “independent” clinical decisions, and providing students with the opportunity to receive immediate feedback from both the standardized patient and faculty, giving them the opportunity to process and reflect upon the encounter.

### Limitations

The curriculum has a few limitations that need to be considered in the implementation. First, it is costly in terms of standardized patient and staff time. Second, limited faculty availability to observe the encounters often hinders the provision of immediate feedback to students. Ideally, it would be desirable to have each follow-up patient scenario reflect students’ individual clinical decision-making.

Evaluation results are subject to a few limitations. The model was implemented at a single medical school and evaluated with one class year, generating a small sample size. Additional evaluation and implementation at other institutions are needed to fully determine the effectiveness of the model for providing continuity experience and teaching clinical management of chronic diseases. Student self-reported confidence levels may not be reflective of actual knowledge and ability to perform in a clinical setting. Future studies should consider a more objective, standardized means of measuring student educational outcomes.

## Conclusion

This series of standardized patient encounters offers a promising approach to providing students with experience in continuity of care and clinical disease management in a longitudinal setting. The model supports other clinical experiences in medical schools and provides a standardized method for teaching chronic disease management and continuity relationships, conferring opportunities for student learning not always available in real-world clinical settings. In the future, other medical schools might consider adapting this model for their own institutions. The model also has promise for interprofessional education, by incorporating students from other disciplines, such as nursing and physical therapy, and having medical students make appropriate referrals. In light of the growing problem of finding clinical training sites, particularly in primary care settings [13], this simulated model offers an innovative solution to providing medical students with clinical experience focused on the continuity relationship.

## Additional files

Additional file 1: L-OSCE Pre-Test- Final. (DOCX 22 kb) Additional file 2: L-OSCE Post-Test- Final. (DOCX 22 kb)

## Abbreviations

EMR: electronic medical record
IRB: Institutional Review Board
SD: Standard deviation
