# Supplementary material for: Using standardized patient encounters to teach longitudinal continuity of care in a family medicine clerkship
Source: BMC Med Educ. 2016 Aug 17;16:208. doi: 10.1186/s12909-016-0733-y (PMC4989459; doi:10.1186/s12909-016-0733-y)
Supplement: Additional file 1: — L-OSCE Pre-Test- Final. (DOCX 22 kb) [file 12909_2016_733_MOESM1_ESM.docx]

| 1. Please rate your previous experience (exposure either in course work or clinical settings) with the following : | | | | |
| --- | --- | --- | --- | --- |
|  | No experience | Little experience | Moderate experience | Lots of experience |
| Providing continuity of care | 0 | 1 | 2 | 3 |
| Establishing rapport with patients | 0 | 1 | 2 | 3 |
| Expressing empathy | 0 | 1 | 2 | 3 |
| Management of *diabetes* | 0 | 1 | 2 | 3 |
| Management of *hypertension* | 0 | 1 | 2 | 3 |
| Management of *hyperlipidemia* | 0 | 1 | 2 | 3 |
| Lifestyle counseling on *smoking* | 0 | 1 | 2 | 3 |
| Lifestyle counseling on *diet* | 0 | 1 | 2 | 3 |
| Lifestyle counseling on *exercise* | 0 | 1 | 2 | 3 |
| Demonstrating whole person care | 0 | 1 | 2 | 3 |
| Understanding how a patient’s context impacts their health | 0 | 1 | 2 | 3 |
| Using an EMR in conjunction with a patient visit | 0 | 1 | 2 | 3 |

FAMILY MEDICINE CLERKSHIP SURVEY

Date: _______________________ ID #: __________________________________

1. In your medical school experience, approximately how many times have you seen the same patient more than once in an out-patient setting:

○ Never

○ Once

○ 2- 3 times

○ More than 3 times

○ Not applicable

1. Which third-year clerkships have you had so far (check all that apply):

○ Family Medicine ○ Psychiatry

○ Surgery ○ Pediatrics

○ Internal Medicine

○ OB/GYN

| 1. Please rate your **confidence** in your ability to successfully do the following : | | | | |
| --- | --- | --- | --- | --- |
|  | Not at all confident | A little confident | Moderately confident | Very confident |
| Provide continuity of care | 0 | 1 | 2 | 3 |
| Establish rapport with a patient | 0 | 1 | 2 | 3 |
| Express empathy | 0 | 1 | 2 | 3 |
| Management of *diabetes* | 0 | 1 | 2 | 3 |
| Management of *hypertension* | 0 | 1 | 2 | 3 |
| Management of *hyperlipidemia* | 0 | 1 | 2 | 3 |
| Conduct lifestyle counseling on *smoking* | 0 | 1 | 2 | 3 |
| Conduct lifestyle counseling on *diet* | 0 | 1 | 2 | 3 |
| Conduct lifestyle counseling on *exercise* | 0 | 1 | 2 | 3 |
| Demonstrate whole person care | 0 | 1 | 2 | 3 |
| Understand how a patient’s context impacts their health | 0 | 1 | 2 | 3 |
| Use an EMR in conjunction with a patient visit | 0 | 1 | 2 | 3 |

1. What does continuity of care mean to you? Do you believe there are benefits (for the doctor, the patient and/or the health care system) when continuity of care is provided? Why or why not?

_________________________________________________________________________________________________________________________________________________________________________________________________________________________________________________________________________________________________________________________________________________________________________________________________________________________________________________________________________________________________________________________________________________________________________________________________________________________________________________________________________________________________________________________________________________________________________________________________________________________________________________________________________________________________________

Thank you!
