# Supplementary material for: Using standardized patient encounters to teach longitudinal continuity of care in a family medicine clerkship
Source: BMC Med Educ. 2016 Aug 17;16:208. doi: 10.1186/s12909-016-0733-y (PMC4989459; doi:10.1186/s12909-016-0733-y)
Supplement: Additional file 2: — L-OSCE Post-Test- Final. (DOCX 22 kb) [file 12909_2016_733_MOESM2_ESM.docx]

FAMILY MEDICINE CLERKSHIP Post

Date: _______________________ ID #: _______________________________

| 1. After participating in the Longitudinal OSCE (L-OSCE), please rate your **confidence** in your ability to successfully do the following : | | | | |
| --- | --- | --- | --- | --- |
|  | Not at all confident | A little confident | Moderately confident | Very confident |
| Provide continuity of care | 0 | 1 | 2 | 3 |
| Establish rapport with a patient | 0 | 1 | 2 | 3 |
| Express empathy | 0 | 1 | 2 | 3 |
| Management of *diabetes* | 0 | 1 | 2 | 3 |
| Management of *hypertension* | 0 | 1 | 2 | 3 |
| Management of *hyperlipidemia* | 0 | 1 | 2 | 3 |
| Conduct lifestyle counseling on *smoking* | 0 | 1 | 2 | 3 |
| Conduct lifestyle counseling on *diet* | 0 | 1 | 2 | 3 |
| Conduct lifestyle counseling on *exercise* | 0 | 1 | 2 | 3 |
| Demonstrate whole person care | 0 | 1 | 2 | 3 |
| Understand how a patient’s context impacts their health | 0 | 1 | 2 | 3 |
| Use an EMR in conjunction with a patient visit | 0 | 1 | 2 | 3 |

| 2. How would you rate the L-OSCE: | | | | | |
| --- | --- | --- | --- | --- | --- |
|  | Poor | Fair | Neutral | Good | Excellent |
| As a simulation of typical family medicine practice |  |  |  |  |  |
| As a simulation of chronic disease management |  |  |  |  |  |
| As a simulation of continuity of care |  |  |  |  |  |
| As representative of the type of patients seen in family medicine practice |  |  |  |  |  |
| As an important contribution to your medical education |  |  |  |  |  |

3. During the Family Medicine clerkship, how many times did you see the same patient more than once in an out-patient setting?

○ Never

○ Once

○ 2- 3 times

○ More than 3 times

○ Not applicable

4. To what extent can you apply the feedback provided during the L-OSCE to your clinical rotations?

Not at all A Little A Lot

5. Which specific skills or knowledge did you feel you gained from this experience that could be applied to real patient encounters?

____________________________________________________________________________________________________________________________________________________________________________________________________________________________________________________________________________________________________________________________________________________________________________________________________________________________________

6. What did you like best about the L-OSCE?

___________________________________________________________________________________________________________________________________________________________________________________________________________________________________________________________________________________________________________________________

7. What suggestions do you have for improving the L-OSCE?

___________________________________________________________________________________________________________________________________________________________________________________________________________________________________________________________________________________________________________________________

8. What does continuity of care mean to you? Do you believe there are benefits (for the doctor, the patient and/or the health care system) when continuity of care is provided? Why or why not?

_________________________________________________________________________________________________________________________________________________________________________________________________________________________________________________________________________________________________________________________________________________________________________________________________________________________________________________________________________________________________________________________________________________________________________________________________________________________________________________________________________________________________________________________________________________________________________________________________________________________________________________________________________________________________________________________________________________________________________________

Thank you!
